# Convergence of longevity and immunity: lessons from animal models

**DOI:** 10.1007/s10522-019-09801-w

**Published:** 2019-02-22

**Authors:** Jingnu Xia, Maria Gravato-Nobre, Petros Ligoxygakis

**Affiliations:** Laboratory of Cell Biology, Development and Genetics, Department of Biochemistry, University of Oxford, South Parks Rd, Oxford, OX1 3QU UK

**Keywords:** Model organisms, Immunity, Ageing, Microbiome, Gut, Brain

## Abstract

An increasing amount of data implicate immunity-mostly innate immunity-in the ageing process; both during healthy ageing as well as in neurodegenerative diseases. Despite the aetiology however, the underlying mechanisms are poorly understood. Here we review what we know from model organisms (worms, flies and mice) on the possible mechanistic details that connect immunity and ageing. These links provide evidence that inter-tissue communication (especially the interaction between gut and brain), hormonal control mechanisms and intestinal microbiota determine immune system activity and thus influence lifespan.

## Introduction

Pro-inflammatory immune responses are our first line of defence against infectious non-self. Inflammation however, has a cost. During the life-history of a human, low-grade inflammation, develops gradually and contributes to the pathogenesis of a range of age-related diseases from leaky gut to neurodegeneration. Conversely, ageing through cell senescence, can influence immune function with the depletion of the pool of naïve T-cells ready to respond to infection making older individuals more vulnerable to viral disease and less responsive to vaccination regimes. This can in turn, influence human lifespan. In the apparent complexity of this dual relationship it is difficult to arrive at a mechanism of causality because cause and consequence are intimately linked.

In the quest for mechanisms however, simpler genetically tractable organisms are our allies. It holds also true for animals like the nematode worm *Caenorhabditis elegans*, the fruit fly *Drosophila melanogaster* or the mouse *Mus musculus*, that organismal lifespan and immune activation are interlinked. The advantage here is that genetic manipulations could reveal how immunity influences lifespan and how longevity modifies resistance to infection. In this review, we will briefly summarise where the stage our knowledge is currently at, on the interplay between longevity and immunity based on work in these three model systems.

## *Caenorhabditis elegans*

*Caenorhabditis elegans* has served as an attractive invertebrate model system to study the molecular and cellular aspects of conserved biological processes such as infection, disease pathogenesis and ageing. Notable examples linking immune activation to lifespan-regulation include evolutionarily conserved pathways such as the insulin-like growth factor-1 signalling (IIS), two key transcriptional regulators of environmental stress namely the heat shock response factor-1 (HSF-1) and the hypoxia inducible factor 1 (HIF-1), the p38 mitogen-activated protein kinase (MAPK) and autophagy. In most cases genetic perturbations that diminish cytoprotective programs within the cell reduce the organismal susceptibility to pathogen infection (Alper et al. 2010). However, exceptions to this appear to be the genes in the IIS pathway. In worms, the insulin receptor DAF-2 (homolog of IGF-1), DAF-16 (homolog of FOXO transcription factors) and AGE-1 (homolog of PI3K) are three critical components of IIS and important regulators of ageing and immunity. Reducing or silencing DAF-2 in worms can extend lifespan but also increase resistance to infections by *Bacillus subtilis*, *Coxiella burnetii*, *Enterococcus faecalis*, *Staphylococcus aureus* and *Pseudomonas aeruginosa* (Garsin et al. 2003). This resistance is completely dependent on DAF-16 as lack of this protein suppresses both lifespan extension and pathogen resistance. In addition, ablation of the nematode germline, either physically using a laser or genetically using sterilizing mutations, leads to increased nematode lifespan. The timing of DAF-16-dependent gene activation in non-fertile mutant worms coincides with the onset of embryonic development in wild-type animals, suggesting that signals from developing embryos normally downregulate the immune response of their parents (Miyata et al. 2008). Reproduction has also been found to suppress immunity in flies, where mating can suppress the immune system through hormonal signalling (Schwenke and Lazzaro 2017).

Under certain temporal conditions, the pathogen resistance of non-fertile nematodes is completely dependent on *daf*-*16* (Evans et al. 2008) but in other cases this is dependent on MAPK but not DAF-16 (see Alper et al. 2010). Like DAF-16, the heat shock factor HSF-1 transcription factor, is repressed by insulin signalling. HSF-1 functions with DAF-16 to regulate proteostasis and chaperone expression when active in response to heat stress (Singh and Aballay 2006; Morton and Lamitina 2013).

### Gut

In many ways, the *C. elegans* gut represents the prototypical intestine: a feeding tube that consumes bacteria in rotting vegetation or in the soil. Since these substrates are rife with opportunistic pathogens the gut needs to withstand exceptional levels of stress given its inability to regenerate. Therefore, the coupling of increased lifespan and resistance to infection appears to be a common theme. To underline this point, one needs to note the declining activity of the MAPK pathway in the intestine during ageing, which increases susceptibility to infection and reduces life expectancy (Youngman et al. 2011). Overexpression of *hsf*-*1* promotes longevity and delays age-related protein misfolding and proteotoxicity while it also plays important roles in *C. elegans* innate immunity where it functions in the intestine to inhibit pathogen-induced protein aggregation and induce resistance against *P. aeruginosa* (Singh and Aballay 2006). Loss of HIF-1 or its target namely, the cytochrome P450 gene, *cyp*-*36A1* regulate non-autonomously the nuclear receptor NHR-46 in the intestine to promote longevity as well as resistance to *P. aeruginosa* (Pender and Horvitz 2018). Moreover, this HIF-1-dependent endocrine signalling, puts forward the notion of inter-tissue communication in controlling behaviour and immunity with the gut playing a prominent role as a major communication hub. Finally, studies in *C. elegans* have shown the connection between autophagy gene expression and host tolerance towards fungal pathogens such as microsporidia (Troemel et al. 2008) while intestinal-specific autophagy regulates longevity in dietary restricted worms (reviewed in Kuo et al. 2018). Moreover, intestinal epithelial cells recognize stress and prompt avoidance (Melo and Ruvkun 2012), one of the first defences of this model host as well as in other animals including humans (reviewed in Curtis 2014).

### Brain

*Caenorhabditis elegans* provides a unique opportunity to unravel the mechanisms by which neuronal aging and intestinal immunity are orchestrated. Similar to mammals, neuronal signalling in *C. elegans* depends on an array of small molecule neurotransmitters (see Bargmann 1998). Recently, the insulin-like neuropeptide encoded by *ins*-*11* has been shown to play a role in avoidance of *P. aeruginosa* (Lee and Mylonakis 2017). The transcriptional expression of *ins*-*11* is controlled through transcription factor *hlh*-*30* and the MAPK pathway. *ins*-*11* negatively controls signal pathways in neurons that regulate aversive learning behaviour. Attenuation of *ins*-*11* increased avoidance behaviour and survival on pathogenic bacteria but decreased opportunities to find a food source as well as lowered energy storage and the number of viable progeny (Lee and Mylonakis 2017). Both ageing and infection provoke neuromuscular changes as well as mobility and pharyngeal pumping decline. In addition, examination of neuronal morphology in *C. elegans* has revealed aberrant neurite branching and structures along the neuronal processes as well as age-associated synaptic deterioration (Tank et al. 2011; Pan et al. 2011; Toth et al. 2012).

### Microbiome

One relatively unexplored aspect of the nexus between ageing and immunity is the microbiome (reviewed in O’Toole and Jeffery 2015). Although there is no consensus on whether the *C. elegans* microbiome is analogous to that of humans, the worm should be an ideal model to identify and study evolutionary conserved gut-microbe interactions since it lives in and feeds on bacteria and has developed behaviours to avoid infectious agents. The issue until now was that there has been no survey of the “representative” microbiota of *C. elegans* in the wild. Recent studies have mitigated this, finding bacterial species, which colonise and associate stably with the worm intestine in the wild, including some commensal *Pseudomonas* strains with anti-fungal properties (Dirksen et al. 2016; Berg et al. 2016; Samuel et al. 2016). The next step will be to study how different mixtures of these bacteria modulate neuronal and intestinal signalling, immune responses and ageing. In parallel, systematic screens with mutants of the *E. coli* strain K12 used in the laboratory for worm culture has revealed that overproduction of bacterial folate (Virk et al. 2016) and colanic acid (Han et al. 2017) respectively reduces and increases host longevity.

## *Drosophila*

*Drosophila* has been the model of choice to explore innate immunity in the last forty years starting with the seminal work of Hans Boman (Boman et al. 1972). Since then a growing body of evidence has pointed towards the age-dependent decline of the immune response and the adverse effects on lifespan that has any chronic deregulation of antimicrobial gene expression in the absence of infection. Again, the gut (as well as the brain-see below) plays a leading role.

### Gut

Deregulation of the immune deficiency (IMD) NF-κB signalling pathway in the midgut of old animals due to the loss of PGRPs of intracellular negative regulators such as *pirk* lead to gut cell death and a reduced lifespan (Paredes et al. 2011). Moreover, age-dependent inflammation of the fat body (the fly’s equivalent of the mammalian liver) secrete circulating PGRPs that repress IMD activity in the midgut, thereby promoting gut hyperplasia. Further, fat body immunosenescence is caused by age-associated lamin-B reduction specifically in fat body cells, which then contributes to heterochromatin loss and de-repression of genes involved in immune responses. As lamin-associated heterochromatin domains are enriched for genes involved in immune response in both *Drosophila* and mammalian cells this seems to be an evolutionary conserved process of immune senescence (Chen et al. 2014). This work showed that functional immunosenescence also happens in invertebrates. In addition, Nakamura et al. verified that the “senescence-associated secretory phenotype”, or SASP, which is the hallmark of cell senescence in mammals can be displayed by *Drosophila* epithelial tissues where mitochondrial respiratory function is simultaneously downregulated in cells expressing a gain of function form of Ras (Nakamura et al. 2014).

### Brain

In addition to intestinal dysbiosis, chronic immune activity over and above the baseline leads to brain neurodegeneration. Mutants for negative regulators of the IMD (for Immune Deficiency) NF-κΒ pathway predispose flies to early neurodegeneration and a short lifespan (Kounatidis et al. 2017). When the activity of Relish, the NF-κΒ orthologue is blocked in glia of these mutants, both neurodegeneration and lifespan reduction are rescued (Kounatidis et al. 2017). This puts forward glia as an important tenant of neurodegeneration in flies as has been shown in mice and humans (see below). In the latter, genome-wide association studies have correlated largely microglia-expressed genes of the innate immune system as risk factors of late on-set Alzheimer’s (International Genomics of Alzheimer’s Disease Consortium 2015). In addition, silencing NF-κΒ in both flies and mice has been shown to extend lifespan in healthy animals through glucagon-dependent hormonal signalling, indicating an evolutionary conserved role for this NF-κΒ/metabolic axis in systemic ageing (Kounatidis et al. 2017 in flies; Zhang et al. 2013 in mice).

### Microbiome

In contrast to *C. elegans* where the study of the microbiome has just started, the fruit fly microbiome has been under intense scrutiny both in lab conditions (for a small selection see Wong et al. 2011; Mistry et al. 2017; Pais et al. 2018) and in the wild (see for example Chandler et al. 2011; Bost et al. 2018). There are some conflicting results on whether the presence of the microbiota is beneficial, but most labs have reported a strong positive effect early on in life with pervasive influence on physiology and metabolism. However, microbiota is connected to adverse effects on longevity later in life (Clark et al. 2015). Studies in laboratory settings have associated specific microbiota compositions with fly age where generally, the presence of *Lactobacteriaceae* in a relatively simple but diverse microbiome signifies younger ages while a significantly increased relative abundance of *Acetobacteriaceae* and low variability denotes aged individuals (Wong et al. 2011; Clark et al. 2015; Mistry et al. 2017). Therefore, like in humans there is a loss of diversity associated with chronological ageing (reviewed in O’Toole and Jeffery 2015). This phenotypic transition of the fly microbiome during healthy ageing can be perturbed by immune deregulation, which results in dysbiosis (Ryu et al. 2008). Loss of Peptidoglycan Recognition Proteins (PGRPs), with an enzymatic activity against bacterial Peptidoglycan leads to inability in decreasing the immunogenicity of the intestinal bacterial flora and causes a short lifespan (Paredes et al. 2011; Guo et al. 2014). Moreover, flies unable to intracellularly suppress NF-κΒ signalling and anti-microbial peptide gene induction also become short-lived with a constantly inflamed gut and a high degree of enterocyte death (Paredes et al. 2011; Fernando et al. 2014). Of note, there is an age-dependent reduction of intestinal barrier function in healthy flies (Rera et al. 2014). In addition, mutant flies with loss of septate junction organisation are predisposed to increased gut “leakiness” and a reduced lifespan. This phenotype can be rescued using antibiotics indicating that it is microbiota-dependent (Bonnay et al. 2013).

### Macrophages

In healthy flies, susceptibility to infection increases with age as seen by measurements of antimicrobial peptide gene induction and host survival. When the phagocytic receptor *croquemort* is missing, flies are exposed to chronic IMD activity leading to a reduced lifespan (Guillou et al. 2016). Moreover, there is an age-dependent decline of phagocytic ability as well as impairment in the processing of phagocytosed vesicles in older flies indicating an immunosenescent phenotype in older flies (Mackenzie et al. 2011; Horn et al. 2014).

## Mice

In contrast to the previous two model animals, mice have both innate as well as adaptive immunity underlined by clonally expanded memory cells.

### Gut

Compromised intestinal barrier function in humans has been associated with diseases such as Intestinal Barrier Disease (IBD), Intestinal Barrier Syndrome (IBS) and Crohn’s disease (reviewed in Odenwald and Turner 2017). Changes in the permeability of the mouse gut, which results in “leaky gut” has consequences on health span as demonstrated by mice with a knock-out in the main component of intestinal mucin that develop colitis (Van der Sluis et al. 2006). In this context, increased age-associated levels of Tumour Necrosis Factor (TNF) have a negative impact on gut permeability and impacts on lifespan while *IL*-*10*^−*/*−^ knock-out mice have (along with their immune defects) increased intestinal permeability and develop early colitis compromising health span and lifespan (Odenwald and Turner 2017). In contrast, TNF-deficient mice are protected from age-associated inflammation (Thevaranjan et al. 2017).

Along with its barrier role, gut-brain communication is also an important aspect of intestinal function. The vagus nerve, provides the primary parasympathetic control of basic intestinal functions, with abundant innervation of the stomach, small intestine, and appendix, terminating before the distal colon (Hopkins et al. 1996). Stimuli in the intestine can trigger vagal afferent signalling, which is a critical component of neuroimmune inflammatory reflex circuits that contribute to tonic peripheral immune regulation (Pavlov and Tracey 2015). Evidence suggests that the vagus nerve may act as a direct conduit by which material from the intestine can pass to the brain in humans (Pomfrett et al. 2007) and rats (Holmqvist et al. 2014). In this context, it has been proposed that intestinal inflammation maybe carried to the brain to initiate neurodegeneration and more specifically Parkinson’s Disease (PD) (reviewed in Houser and Tansey 2017). Mice that express human α-synuclein (αSYN) in their gut can develop PD in the brain through transferring αSYN, but not if the vagus nerve is disrupted (Luk et al. 2012). In turn, this may explain some of the non-neuronal intestinal symptoms of Parkinson’s such as constipation and hyposmia, which appear long before any neurological phenotype (Chen et al. 2015; Park et al. 2015).

### Brain

There is now increasing evidence that inflammation regulates ageing (reviewed in Franceschi and Campisi 2014). But which tissues contribute to this is less clear. Brain neuroinflammation represents a critical factor contributing to progression of neurodegeneration (reviewed in Glass et al. 2010). NF-κΒ is the major regulator of inflammation and its sustained activation in forebrain neurons elicits a selective inflammatory response accompanied by decreased neuronal survival and impaired learning and memory (Maqbool et al. 2013). More recent experiments of transient NF-κΒ activation in astrocytes (a type of microglia) through a diverse array of inflammatory cues (infection or application of pro-inflammatory cytokines), resulted in non-cell autonomous neurodegeneration (Lattke et al. 2017). The central position of microglia innate immunity in neurodegeneration and especially in the risk for late on-set Alzheimer’s Disease (AD) is exemplified in human genome-wide association studies (International Genomics of Alzheimer’s Disease Consortium 2015). This aetiology has been confirmed in humanised mice expressing the risk factor TREM2 in microglia (Song et al. 2018; Lee et al. 2018). Loss of TREM2 has been associated with increased risk of late on-set AD and increased TREM2 expression in mouse microglia had an anti-inflammatory rescuing effect with the downregulation of several pro-inflammatory markers (Lee et al. 2018). This ameliorated the neuropathological and behavioural deficits of AD mouse models (Lee et al. 2018).

As in flies (see Kounatidis et al. 2017), the reverse type of regulation (i.e., downregulating instead of intensifying NF-κΒ activity) increases lifespan and health span through the release of an immune-hormonal axis (Zhang et al. 2013). Reducing NF-κΒ in the microglia of the hypothalamus releases the gonadotropin-releasing hormone (GnRH). This restoration decelerates ageing showing that the hypothalamus has a programmatic role in ageing via immune-neuroendocrine integration (Zhang et al. 2013). Moreover, modulating NF-κB activity in astrocytes identifies the significant roles this pathway plays in regulating blood glucose, blood pressure and body weight three important indicators of health across the life course (Zhang et al. 2017). Overnutrition increases NF-κΒ activity in astrocytes and decreases their plasticity. This may underlie early effects that could result in the later pathology of diseases such as obesity, increasingly see as a condition of chronic inflammation (reviewed in Saltiel and Olefsky 2017).

### Microbiome

Germ-free mice live almost 100% to 600 days in contrast to their conventionally-reared counterparts that reach this point with a 60% survival probability (Thevaranjan et al. 2017). In addition, germ free mice do not display age-associated inflammation while their macrophages retain their antimicrobial activity (Thevaranjan et al. 2017). This indicates that age-associated changes of the microbiota are a significant driver of lifespan where TNF-mediated inflammation acting as an effector of morbidity. Indeed, treatment of mice with anti-TNF antibodies reversed age-associated changes in the microbiota and ameliorated life expectancy (Thevaranjan et al. 2017). Therefore, reversing these age-related microbiota changes represents a potential strategy for reducing age-associated inflammation and the accompanying morbidity (Thevaranjan et al. 2017).

### T and B lymphocytes

These cells undergo immune senescence. Senescence is age-dependent and is the driving force for immune ageing. During ageing, both T and B cells will be depleted and the memory B cells, long-lived plasma cells and peripheral T-cells show defects. In addition, the provision by the thymus of naïve T-cells for the adaptation to new pathogens is limited. The mechanisms of these age-related defects are not fully elucidated but reduced autophagy, is a major driving force for immune senescence. In murine T cells, neutrophils and macrophages, autophagy is attenuated during ageing and autophagy-deficient cells display premature ageing traits. Signalling pathways, which control autophagy in immune cells are NF-κB, which can activate autophagy or lysosome genes and PI3K, which is associated with the formation of the auto-phagophore (reviewed in Zhang et al. 2016).

## Conclusion

**Fig. 1:**
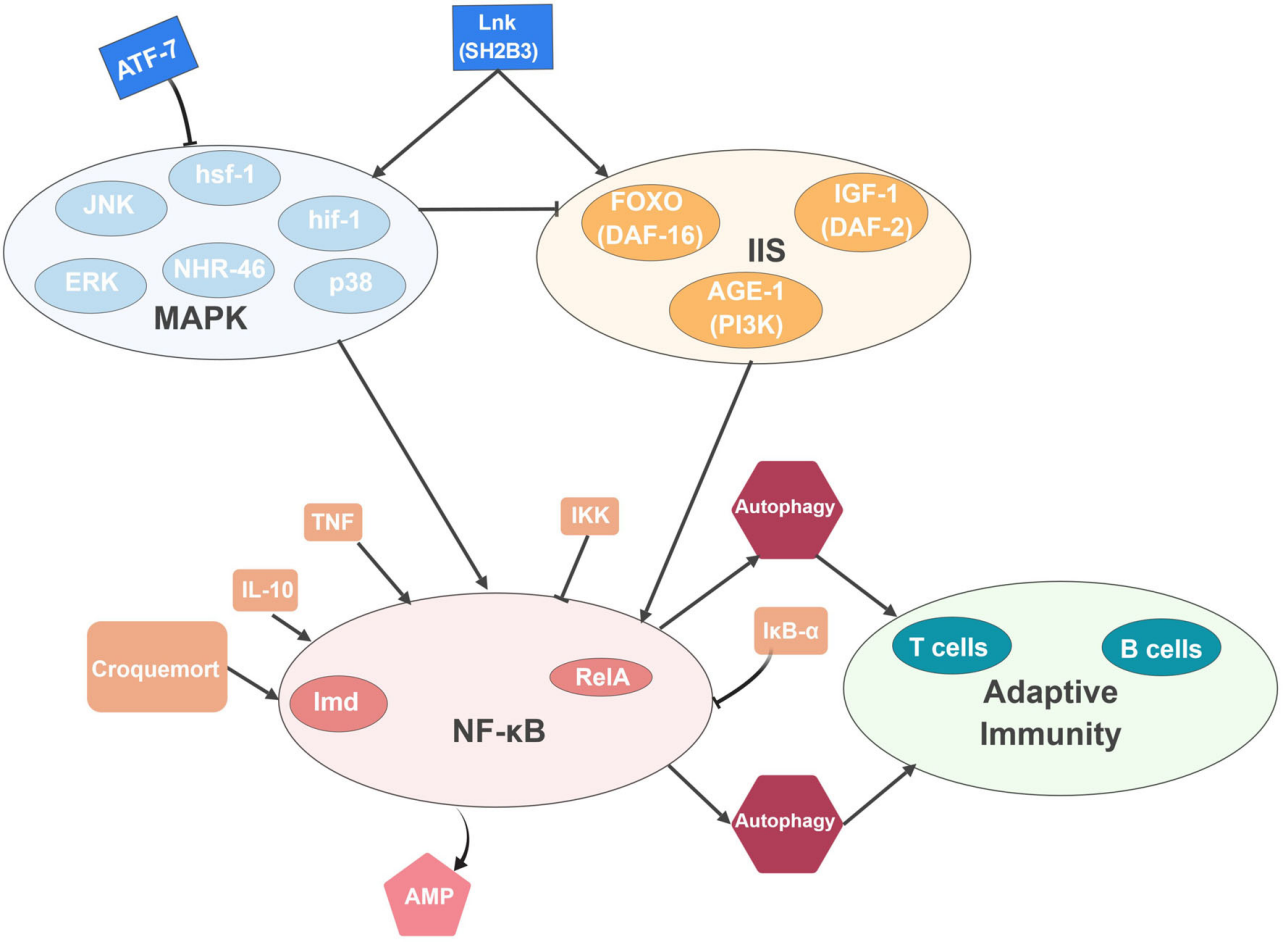
Where longevity and immunity converge A summary of signalling pathways and cells with a documented role on the nexus of immunity and longevity (see main text for details). MAPK signalling has an important role in regulating immunity and lifespan in worms and flies, IIS in *C. elegans*, NF-κB in flies (Croquemort, Imd, AMPs) and mice (TNF, IL-10, IKK, RelA) and autophagy in all three animals. Since worms and flies are devoid of adaptive immunity, the interaction of autophagy and T- and B cell based responses, concerns only mice

Although one cannot directly extrapolate the above findings to humans, model organisms have been valuable discovery tools to explore the processes underlying age-dependent disorders and separate between the genetic causes and the physiological consequences in age-related biology. Figure 1 summarises these connections and demonstrates the potential for evolutionary conservation of the underlying mechanisms. More work is needed to identify all common patterns in the data, but model organisms already point to a “direction of travel”. Namely, that gut-brain interactions and the microbiome, which may modulate these interactions are some of the factors that influence systemic ageing often through immune signalling pathways. Thus, immune activity and life expectancy seem to be intricately linked.
